# Measuring the unseen: mobilizing citizen scientists to monitor groundwater in Nepal

**DOI:** 10.1007/s10661-021-09265-x

**Published:** 2021-08-05

**Authors:** Rajaram Prajapati, Rocky Talchabhadel, Bhesh Raj Thapa, Surabhi Upadhyay, Amber Bahadur Thapa, Brandon Ertis, Jeffrey C. Davids

**Affiliations:** 1 Smartphones For Water Nepal (S4W-Nepal), Lalitpur, Nepal; 2 Texas A&M AgriLife Research, Texas A&M University, El Paso, TX USA; 3 Universal Engineering and Science College, Lalitpur, Nepal; 4 Smartphones4Water, Chico, USA; 5 California State University, Chico, USA

**Keywords:** Groundwater monitoring, Citizen science, Kathmandu Valley, Nepal

## Abstract

Groundwater-level monitoring provides crucial information on the nature and status of aquifers and their response to stressors like climate change, groundwater extraction, and land use changes. Therefore, the development of a spatially distributed long-term monitoring network is indispensable for sustainable groundwater resource management. Despite being one of our greatest unseen resources, groundwater systems are too often poorly understood, ineffectively managed, and unsustainably used. This study investigates the feasibility of establishing a groundwater monitoring network mobilizing citizen scientists. We established a network of 45 shallow monitoring wells in the Kathmandu Valley using existing wells. We recruited 75% of the citizen scientists through personal connections and the rest through outreach programs at academic institutes and site visits. We used various methods to encourage citizen scientists to complete regular measurements and solicited feedback from them based on their experiences. Citizen scientists were more consistent during the monsoon season (June through September) than non-monsoon seasons. The depth-to-water below the ground surface varied from − 0.11 m (negative sign represents a groundwater level higher than the ground surface) to 11.5 m, with a mean of 4.07 m and standard deviation of 2.63 m. Groundwater levels began to rise abruptly with the onset of monsoon season and the shallowest and the deepest groundwater levels were recorded in peak rainfall months and dry months respectively. Citizen science-based groundwater monitoring using existing wells would be an economic and sustainable approach for groundwater monitoring. Improved groundwater-level data will provide essential information for understanding the shallow groundwater system of the valley, which will assist concerned authorities in planning and formulating evidence-based policy on sustainable groundwater management.

## Introduction

Groundwater, which constitutes roughly 98% of the Earth’s freshwater supply, is an important part of the natural water system (Krishan & Rao, 2017). On a global scale, around 2 billion people extract groundwater to fulfil their drinking needs (Morris et al., 2003). Additionally, groundwater is fundamental for irrigated agriculture (Foster & Chilton, 2003; Giordano, 2009; Shah, 2007) and groundwater-dependent ecosystems (Alley et al., 2002; Rohde et al., 2017; Sophocleous, 2002). Groundwater systems are dynamic and primarily influenced by changes in climate, groundwater extraction, and land use (Taylor & Alley, 2001; Thapa et al., 2019). Unfortunately, groundwater is often poorly understood, ineffectively managed, and unsustainably used (Kaur & Rosin, 2007). Overexploitation and contamination are major challenges for sustainable groundwater management. Groundwater management is challenging from both quantity and quality perspectives; characterizing groundwater quality and introducing and spreading contaminants, and estimating groundwater volumes, recharge, and discharge rates are all challenging tasks. Groundwater-level measurements from observation wells are the primary source of information about groundwater systems (Taylor & Alley, 2001), and one of the most practical ways to support sustainable groundwater management is through regular monitoring of groundwater levels (Grieef & Hayashi, 2007). Groundwater-level monitoring provides crucial information on the nature and status of aquifers and their response to climate change, groundwater extraction, and land use variation. Unlike surface water systems, groundwater systems may respond differently to external factors, requiring a distributed network of monitoring wells for sustainable groundwater resource management (Little et al., 2016).

In recent years, the Kathmandu Valley in Nepal (Valley) has experienced increased population and urbanization, which has resulted in scarcity and degraded quality of water (Thapa et al., 2017). Kathmandu Upatyaka Khanepani Limited (KUKL) is a public company responsible for managing and operating the water supply and sanitation system of the Kathmandu Valley. KUKL, the largest municipal water supplier in the Valley, only supplies 115 million liters per day (MLD) in the wet season and 69 MLD in the dry season, which does not meet the current water demand of 370 MLD (KUKL, 2015). This deficit is balanced by water supply through the traditional stone spouts, private dug wells, water tankers, and bottling industries (Thapa et al., 2017). Most people without access to water through KUKL use dug wells, stone spouts, and tube wells to extract groundwater from the shallow aquifer (Thapa et al., 2016). These groundwater sources have been the backbone of water supply in the Valley since ancient times. Uncontrolled population growth coupled with haphazard urbanization has led to excessive extractions of groundwater beyond the rate of sustainable capture (Davids & Mehl, 2015﻿) as well as a reduction in recharge by sealing of ground surfaces through urbanization (Pandey et al., 2010). All of these factors have ultimately resulted in groundwater depletion (Thapa et al., 2019). Overall, groundwater, including both shallow and deep aquifers, consists of about 50% of the total water supply in the Kathmandu Valley (KUKL, 2021). For these reasons, the sustainable use of groundwater resources is a critical issue. Hence, the Government of Nepal has planned to supply an additional 510 MLD water from an inter-basin transfer project called Melamchi Water Supply Project (MWSP) to reduce the dependence on groundwater in the Kathmandu Valley (Thapa et al., 2018). However, there is a lack of reliable scientific information on distribution, discharge, and hydrogeology of shallow groundwater wells, groundwater use scenarios, and the trend of shallow groundwater-level fluctuations (Pandey et al., 2010). This may be due to the absence of a strong institutional mechanism for monitoring and regulating the shallow groundwater system. Furthermore, the available information is scattered among different stakeholders (i.e., several government and non-government organizations, independent researchers, scholars, drilling contractors, etc.) (Pandey et al., 2010).

Creswell et al. (2001) estimated the age and recharge rate of deep groundwater using the radioisotope chlorine-36. The estimated age of deep groundwater was in the range of 200,000 to 400,000 years and the estimated recharge rate was about 5 to 15 mm/year, contributing 40,000 to 1.2 million m^3^/year to the groundwater system. Gurung et al. (2006) conducted geochemical analyses of fluvial-lacustrine aquifer sediments of the Kathmandu Valley to assess the environment for the mobilization of arsenic in groundwater. Chhetri and Smith (1995) performed water quality tests of one hundred wells to analyze the nitrate pollution in groundwater in the Kathmandu Valley and Chitwan. Previous studies have addressed groundwater quantity (e.g., Cresswell et al., 2001; JICA, 1990, Stanley, 1994; Metcalf & Eddy, 2000), groundwater quality (e.g., Khadka, 1993; Chettri & Smith, 1995; Jha et al., 1997; ENPHO, 1999; Gurung et al., 2006), geological formations (e.g., Dongol, 1985; Sakai, 2001; Shrestha et al., 1998; Yoshida & Igarashi, 1984), and hydrogeology (e.g., Keshab, 2003; Metcalf & Eddy, 2000) of the aquifer system of the Kathmandu Valley. Previous studies focused on deep aquifers and their characteristics, whereas there have been a limited number of studies related to the shallow aquifers in the valley.

The Kathmandu Valley Water Supply Management Board (KVWSMB) and the Ground Water Resource Development Board (GWRDB) are responsible for the groundwater monitoring of the Valley. GWRDB presented a comprehensive picture of shallow aquifer mapping of the Kathmandu Valley in 2014 using secondary data. Shrestha and Shah (2014) analyzed the shallow aquifer potential zones by overlaying the thematic maps (geology, aquifer thickness, precipitation, and land use) in terms of weighted overlay methods. However, regular scientific monitoring, through a dense network of monitoring wells, of the groundwater level of shallow wells in the Kathmandu Valley has yet to be carried out (Thapa et al., 2019), and the capacity of the government to install and operate new monitoring wells is constrained by financial and human resources (Little et al., 2016). In this case, the most realistic and economically feasible approach is to utilize existing wells as monitoring wells (Grieef & Hayashi, 2007) and involve local community people as citizen scientists.

Citizen science refers to the participation and collaboration of the citizens (i.e., non-scientists) in scientific research to generate new scientific knowledge, together with professional scientists (Buytaert et al., 2014). The use of citizen science has multiple benefits. From one perspective, citizen science serves as a vital tool to fulfil data gaps in data-scarce areas (Nigussie et al., 2018), whereas, from another, it has many social benefits like increasing scientific literacy, citizen inclusion in local issues, environmental democracy, strengthened governance, and healthier ecosystems (Conard & Hilchey, 2011). The recent development of communication technology along with the growing accessibility of the Internet and smartphones has supported the growth and success of citizen science efforts (Brouwer et al., 2018). Some researches have been performed monitoring water parameters through citizen science such as community-based groundwater monitoring in Rocky View County in Alberta, Canada (Little et al., 2016), West Nose Creek pilot study in Canada (Grieef & Hayashi, 2007), Living Lake Canada (https://livinglakescanada.ca/), and Coastal Groundwater Watch in Bogue bank, North Carolina (Manda & Allen, 2016). The community-based groundwater monitoring program (Little et al., 2016) investigated seasonal and interannual groundwater level and dynamics and their spatial variability in Rocky View County, Canada. Most of the citizen science research works are based in North America and Europe and a few studies have been conducted in South Asia (Walker et al., 2021).

Our working hypothesis was that citizen science-based groundwater monitoring using existing wells would be a feasible option to sustainably generate groundwater level data in an urban center like the Kathmandu Valley with limited resources, where installing new wells solely for monitoring purposes would be difficult due to financial and human resource constraints. The objectives of this study were:

1. To enhance spatiotemporal coverage of shallow groundwater-level monitoring mobilizing citizen scientists.
2. To understand the shallow groundwater-level dynamics of the Kathmandu Valley.
3. To document our citizen science-based groundwater monitoring methodology.
4. To evaluate the pros and cons, and the implementation constraints of the monitoring approach.

### Study area and context

**Fig. 1:**
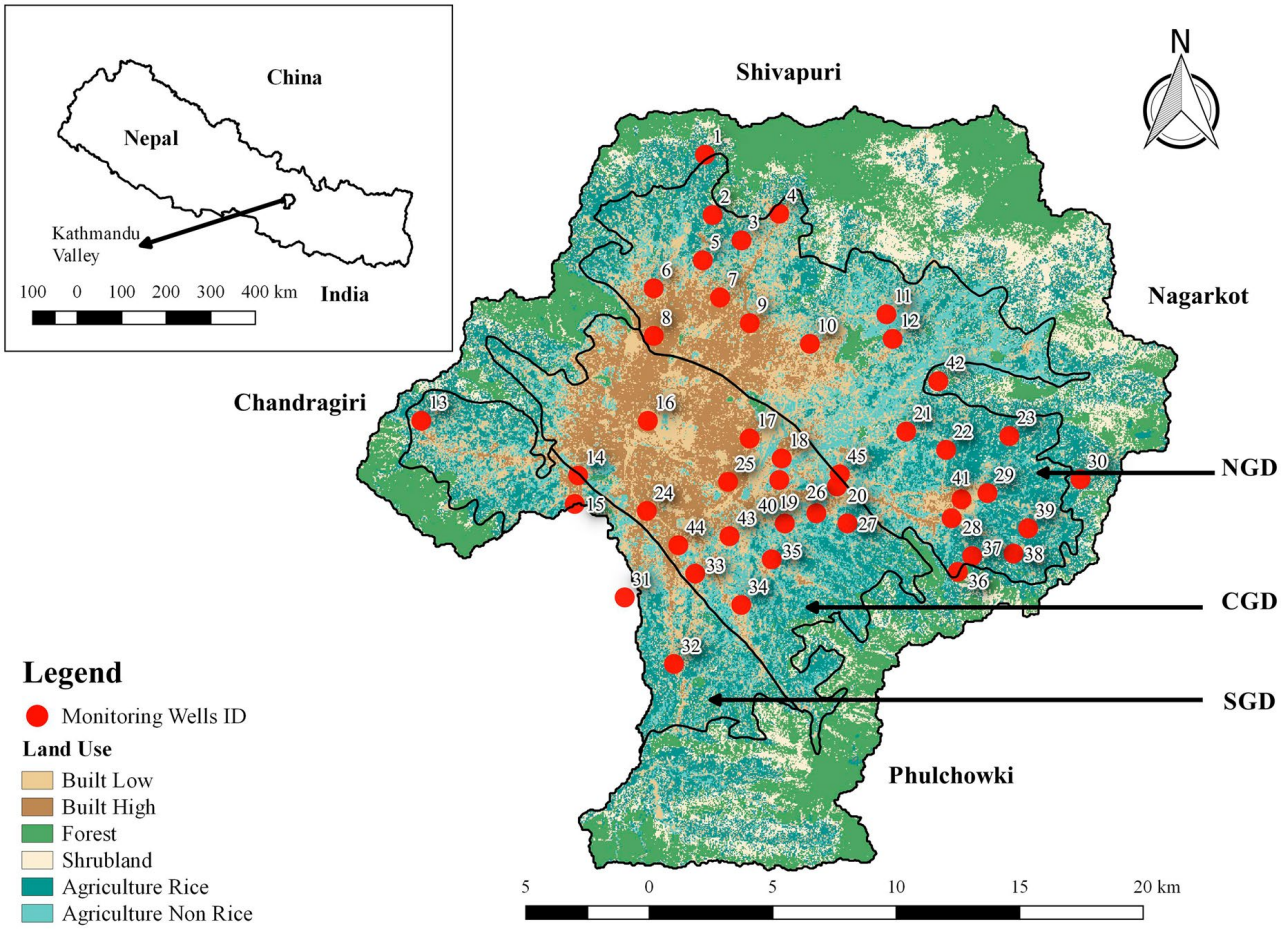
Land use map (Davids et al., 2018) of the Kathmandu Valley watershed with groundwater-level monitoring sites shown as red circles and labeled with numbers. NGD, CGD, and SGD in the figure imply northern, central, and southern groundwater districts, respectively

The Kathmandu Valley lies between 27°32′13″–27°49′10″N latitude and 85°11′31″–85°31′38″ E longitude (Fig. 1). The Kathmandu Valley watershed has an area of approximately 587 km^2^ (Davids et al., 2018). The Valley floor holds the capital city, Kathmandu, and is surrounded by hills: Phulchowki in the southeast, Chandragiri/Champa Devi in the southwest, Shivapuri in the northwest, and Nagarkot in the northeast. It is a roughly circular intermontane basin with an approximate diameter of 25 km and an average altitude of 1350 m above sea level (masl) while the surrounding hills are approximately 2800 masl in elevation (Shrestha et al., 2016). The Kathmandu Valley consists mainly of alluvial plains, alluvial and colluvial fans, fluvial and lacustrine terraces, and steep to very steep sloping mountains. The Köppen-Geiger climate classification of the Valley is Cwa, temperate climate with dry winter (Karki et al., 2016). The climate is mild and generally warm, and there are mainly four seasons depending on the rainfall patterns, namely, (1) pre-monsoon (March – May), (2) monsoon (June – September), (3) post-monsoon (October – November), and (4) winter (December – February) (Talchabhadel et al., 2020).

The floor of the Kathmandu Valley consists of basin fill sediments (quaternary); the surrounding hills are composed of basement rocks (Precambrian to Devonian) and the sediments on the Valley floor are also underlain by basement rocks (Shrestha & Shah, 2014). The Kathmandu Valley is divided into three groundwater districts based on physical and chemical characteristics of the groundwater and geological conditions as seen in Fig. 1 (JICA, 1990). The northern groundwater district consists of unconsolidated highly permeable sand and gravel and has high recharge potential. The central groundwater district consists of very thick (200 m) impermeable black “Kalimati” clay limiting the recharge rate. The southern groundwater district is mostly covered by thick impermeable clay (JICA, 1990).

The approach of citizen science-based monitoring emerged after researchers from Smartphones For Water Nepal (S4W-Nepal), a non-profit research organization in Nepal, mobilized a group of undergraduate students to develop a network of private and public water wells to monitor groundwater levels within Bhaktapur municipality (one of the municipalities in the Kathmandu Valley). Local people gave access to their private wells to measure seasonal groundwater levels for the students. Citizen scientists measured the groundwater levels of 472 dug wells during the 2016 winter (December 2016). We then prepared a water table map that was further used to select 25 representative wells for long-term groundwater-level monitoring. The study showed the significant impact of land use on groundwater-level variations as the built land use area has a deeper groundwater level than agricultural land use. The citizen science approach used in the study seems effective as it was cost-effective, it helped local students understand the status of shallow groundwater table, and it provided a basis for long-term groundwater-level monitoring. The study results only depict the groundwater table of a particular time, and long-term monitoring is crucial to understand the aquifer behavior. The monitoring wells considered were public and private and had high degrees of variability in terms of withdrawal volume and frequency. Motivated by the successful implementation of this small-scale monitoring network, the research team planned to expand the network to the whole Kathmandu Valley. The specific objective was to recruit 45 citizen scientists distributed across the Kathmandu Valley to monitor groundwater levels on a monthly time step to develop a network of shallow monitoring wells and a dataset to support sustainable groundwater resource management.

## Methods

**Fig. 2:**
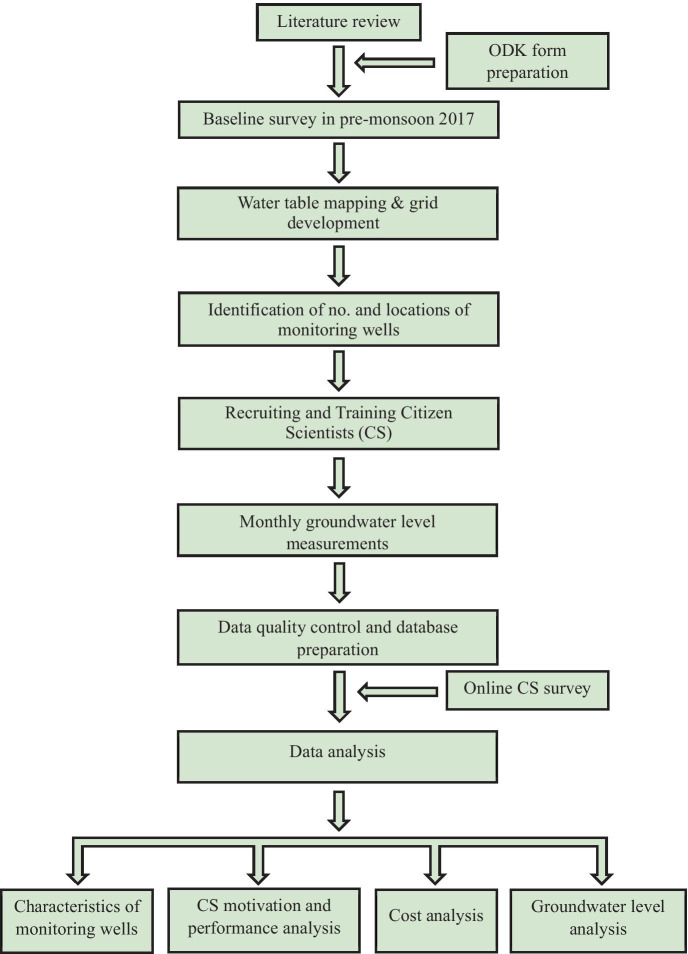
Methodology framework

Figure 2 shows the flowchart of the methodology framework.

### Developing of groundwater monitoring network

#### Baseline survey

A pre-monsoon baseline survey of shallow groundwater wells was conducted in May and June of 2017 across the Kathmandu Valley. Citizen scientists collected groundwater-level data, measurement date and time, geo-locations, overview picture, and water quality data (electrical conductivity (EC) and pH) for approximately 250 public and private wells. EC is a key water quality parameter as it covaries with other water quality parameters, including alkalinity, hardness, and chloride (Kumar & Sinha, 2010). The selection of water quality parameters was based on the availability of water quality meters and instant in situ measurement. The objective of this baseline survey was to develop a water table map of the Valley which could be used to select representative wells for developing a long-term groundwater monitoring network.

#### Grid development and overlay

Using the geospatial software program Quantum GIS (QGIS), a 10 × 10 grid was developed, with dimensions of 3364 m × 3142 m per grid, and overlaid on the watershed map of the Kathmandu Valley. These grids are simply just the division of the entire study area to look into local scale. A total of 73 of 100 grids intersected the watershed. The desired number of monitoring wells was based on one well per grid, resulting in 73 monitoring wells. Since the hills around the edges of the Kathmandu Valley are characterized by dense forests and low population density, the probability of finding existing wells in those grids was very low. As a result, the number of wells for the groundwater monitoring network was reduced to 45 locations, primarily focused on the valley floor.

#### Guidelines for selection of monitoring wells

The following guidelines were utilized for the selection of monitoring wells.

- The well should be easily accessible and groundwater levels should be visible to easily measure the water level via the data collection methods described below.
- Public wells are preferable for ease of access, if there are no data quality constraints.
- Wells with minimal water withdrawals are preferable as the wells with high degrees of variability in terms of withdrawal volume and frequency may not represent true groundwater tables.
- Wells with access constraints (e.g., locked private wells) and/or pumping systems are less preferred.

#### Land use classification

Land use classifications at a 30-m spatial resolution were previously developed using a Landsat 8 satellite image from fall 2015 (October) and ground-based land use observations by citizen scientists (Davids et al., 2018). Three land use types were used to represent the land uses in the Kathmandu Valley: (i) natural land uses (forest, shrubland), (ii) agricultural land uses, and (iii) built land uses. The recharge area contributing to each well (i.e., zone of contribution or ZOC) was assumed to have a radius of 1000 m, and the percentage of different land use types was determined for each well’s ZOC (Prajapati et al., 2021). The land use type of particular well was represented by the land use having a higher percentage in the ZOC.

### Data collection, quality control, and dissemination

#### Data collection

**Fig. 3:**
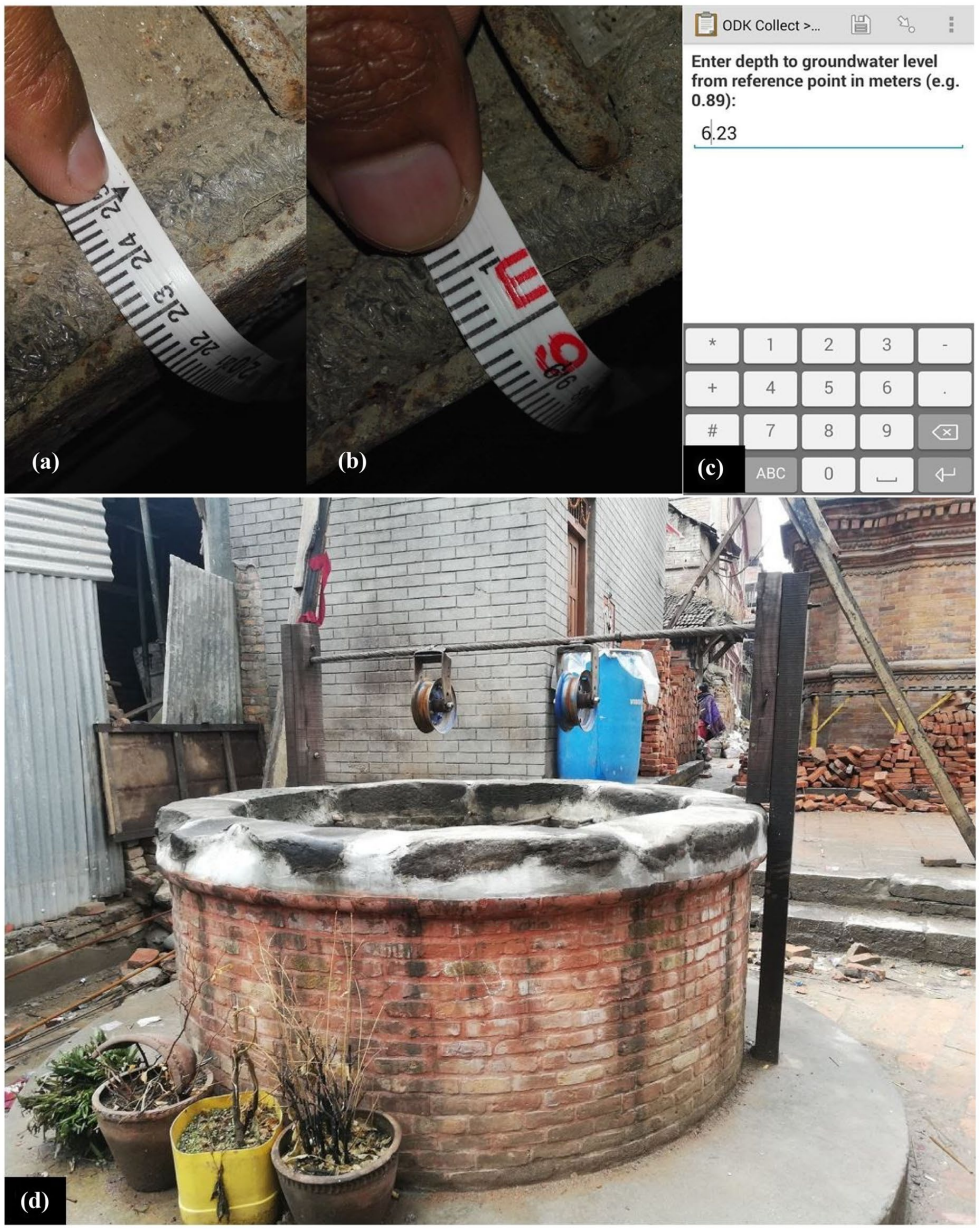
**a** Image of the measuring tape and reference point when the measuring tape just touches the groundwater surface. **b** Image of the measuring tape and reference point when the measuring tape is pulled up so that the next whole meter on the tape is visible. **c** After selecting the parameter to measure, the citizen scientists entered their observations of depth to groundwater level from reference point (m). **d** Image of a monitoring well

Citizen scientists performed groundwater-level measurements using a standard 30-m measuring tape. An Android smartphone application called Open Data Kit Collect (ODK Collect; Anokwa et al., 2009) was used to record and transfer data to the centralized database via Wi-Fi or cellular network. Video tutorials on the installation and use of ODK Collect and groundwater-level measurement procedures are available on S4W’s YouTube channel (https://www.youtube.com/channel/UCrWcJc0rlui6RxGu33xRpGA). The following steps were performed by the citizen scientists for groundwater-level measurements:

- Measurements were performed monthly (on the 15th of the month early in the morning around 7 am before withdrawal of water). As the measurements were performed by several citizen scientists, it is crucial that the data be taken on the same day each month so that the measurements were comparable to each other. Water-level measurement taken just after withdrawal might not represent the actual groundwater table so the measurements were performed before any withdrawal.
- Date, time, and geo-coordinates were recorded using ODK Collect.
- For the first measurement at each monitoring well, ODK Collect was used to record distance to the ground surface from the reference point in meters (m), total depth of well (depth from the reference point to the bottom of well) in meters (m), and circumference of well in meters (m), and an image was taken of the monitoring well.
- The well opening was partially opened, and the measuring tape was lowered from the reference point (i.e., top surface of the casing of well) until the zero-end of measuring tape just touched the groundwater surface.
- Photographs of the measuring tape and reference point when the measuring tape just touched the groundwater surface (Fig. 3a) and when the measuring tape was pulled up so that the next whole meter on the tape was visible (Fig. 3b) were taken, and a numeric reading of depth to groundwater level from the reference point was entered into ODK Collect in meters (m; Fig. 3c).
- The measurement was saved locally to smartphone memory and sent to the S4W-Nepal ODK Aggregate server running on the Google App Engine. ODK Collect was designed to work offline (i.e., without cellular coverage) and can be configured to automatically or manually send data after the coverage is restored.
- Each record was reviewed by S4W-Nepal staff to ensure that the numeric entry from the citizen scientist (Fig. 3c) matches the photographic records of the observation (Fig. 3a, b). Any observed discrepancies were corrected, and records of edits were maintained.

#### Data quality control and dissemination

Manual review of every observation was performed by S4W-Nepal staff for quality control and assurance. This consisted of comparing numeric entries with images and comparing current data points with previous data to check for any inconsistency resulting from human errors. For inconsistent data, citizen scientists were immediately contacted to resolve the error, if any. Figure 3 shows an example of the quality control process for groundwater-level measurements. Final data were manually distributed to interested citizen scientists. A web-based data portal is under development which will allow the public to view and freely download the data to improve the efficiency of data dissemination.

#### Status of monitoring sites

The online survey of 28 citizen scientists conducted in December 2018 – January 2019 included questions on the history of the monitoring well, methods of groundwater extraction, use of extracted groundwater, nearby land uses, and water supply status in the locality of monitoring well. The monitoring wells were classified as either public or private. The major source of water supply in the locality of monitoring sites was also identified.

### Recruitment and training of citizen scientists

Citizen scientists are foundational to the success of any citizen science project; their dedication to consistent, accurate data collection is what will make a project successful. Since groundwater-level monitoring projects typically require years, rather than months, of data collection, the long-term commitment of local citizen scientists is a key component for these projects. Our goal was to conduct shallow groundwater-level monitoring from July 2017 to December 2018 with interested citizen scientists encouraged and supported in continuing data collection after December 2018. In this study, citizen scientists were recruited by various methods including leveraging personal relationships, posts on social media, outreach programs at schools/colleges, and random site visits (Davids et al., 2019). We collaborated with several academic institutes in the Kathmandu Valley to recruit citizen scientists to fulfil the vacant grids of the monitoring network. The outreach programs typically included presentations about the current water scenario of the Kathmandu Valley and S4W-Nepal’s effort on hydrological monitoring in the present context. We also conducted training on groundwater-level measurement and the use of ODK. After trying our best to fulfil all the grids from the abovementioned methods, there were still some vacant grids. For that, we made random site visits to the desired locations, talked with local people, and explained our project to them. Then, with the help of local community members, we found suitable wells for our monitoring purposes and recruited and trained citizen scientists who had functioning smartphones and proper access to the monitoring well. After initial contact and field training, citizen scientists were asked to submit their personal details (name, address, contact, etc.) and monitoring well description (as described in the section “Data collection, quality control and dissemination”).

### Communication and education (motivation)

Recruitment of citizen scientists is important, and their continued dedication is vital for the success of citizen scientists–driven projects (Conrad & Hilchey, 2011; Cooper et al., 2007; Evans et al., 2005). Regular two-way communication between the study team and citizen scientists plays a crucial role in citizen science projects. Motivating citizen scientists is an essential but challenging task. We used various methods to encourage citizen scientists to complete regular measurements such as reminder SMS/follow-up calls before the measurement dates, distributing a thank you message and summary of monthly groundwater-level change through SMS after measurement, providing certificate of volunteering, giving participants access to data, organizing workshops, and offering payment (Davids et al., 2019). Additionally, we created a Facebook group to facilitate follow-up and respond to any queries or problems citizen scientists had. In addition, we published a quarterly newsletter to disseminate information on trends/use of data collected by citizen scientists and provide an update on S4W-Nepal’s recent activities. Finally, an online survey was conducted in December 2018 – January 2019 to solicit feedback from citizen scientists participating in the project. Questions included in the survey asked how they became citizen scientists, whether they wanted to continue volunteering for another 3 years, what motivated them to participate, what aspects of the project they liked/disliked, and if they had any further comments/suggestions for the study team.

### Citizen scientists’ participation

The performance of citizen scientists was analyzed based on regularity and punctuality. Months were categorized into four seasons: (i) monsoon (July – September), (ii) post-monsoon (October – December), (iii) winter (January – March), and (iv) pre-monsoon (April – June). A heatmap of groundwater measurements per season for all citizen scientists depicting the regularity of measurement was developed. Ideally, citizen scientists would measure groundwater levels on the 15^﻿th^ of the month. However, for various reasons, the measurement date varied from the middle to the end of the month. The punctuality of citizen scientists was also analyzed by developing a heatmap with the date of measurements of each citizen scientist in different months.

### Quantification of costs

Quantifying the cost of a citizen science-based groundwater monitoring network is complicated; for simplicity, the cost was estimated as the initial configuration and long-term operational costs (Little et al., 2016). Initial configuration costs included working staff hours for recruiting and training of citizen scientists (including travel cost) and the cost of 30-m measuring tapes. In addition, citizen scientists used their own smartphones for data collection. Long-term operation costs consisted of small financial incentives for citizen scientists, maintenance cost of measuring tape, annual workshop expense, and working staff hours to follow-up with citizen scientists for regular measurement.

### Groundwater level and dynamics

Out of 45 monitoring sites, only 35 monitoring sites had regular (wells having more than 70% measurements) groundwater-level measurements from July 2017 to December 2018 and were included in the final dataset for analysis. The monthly groundwater-level changes of all monitoring sites were analyzed by preparing a heatmap of depth-to-water from the ground surface. The distribution of depth-to-water from the ground surface for all monitoring sites was evaluated by preparing a box plot. Seasonal maps were prepared using QGIS.

## Results and discussion

### Development of groundwater monitoring network

In total, 45 monitoring wells in the network were contained in 28 of the 73 grids. Of the 28 grids, 19 included one well, five included two wells, one included three wells, two included four wells, and one included five wells. Although one monitoring well (ID 31) lay outside the watershed boundary, it was included in the network since it was in an adjacent area still hydrogeologically connected to the Kathmandu Valley and no other monitoring wells were identified in that grid.

A total of 33% of the Valley was classified as natural land use, 41% was classified as agricultural land use, and 26% was classified as built land use (Davids et al., 2018). Nearly half of the 73 grids were either natural land use or more than 50% outside of the watershed boundary and were not expected to have existing monitoring wells. As expected, there were no wells in grids that were a majority natural land use; 25 wells were in built land use and 20 wells were in agricultural land use. The monitoring network was limited to the Valley floor as there were no existing wells in the surrounding mountains which are dominated by natural land use.

### Status of monitoring sites

**Fig. 4:**
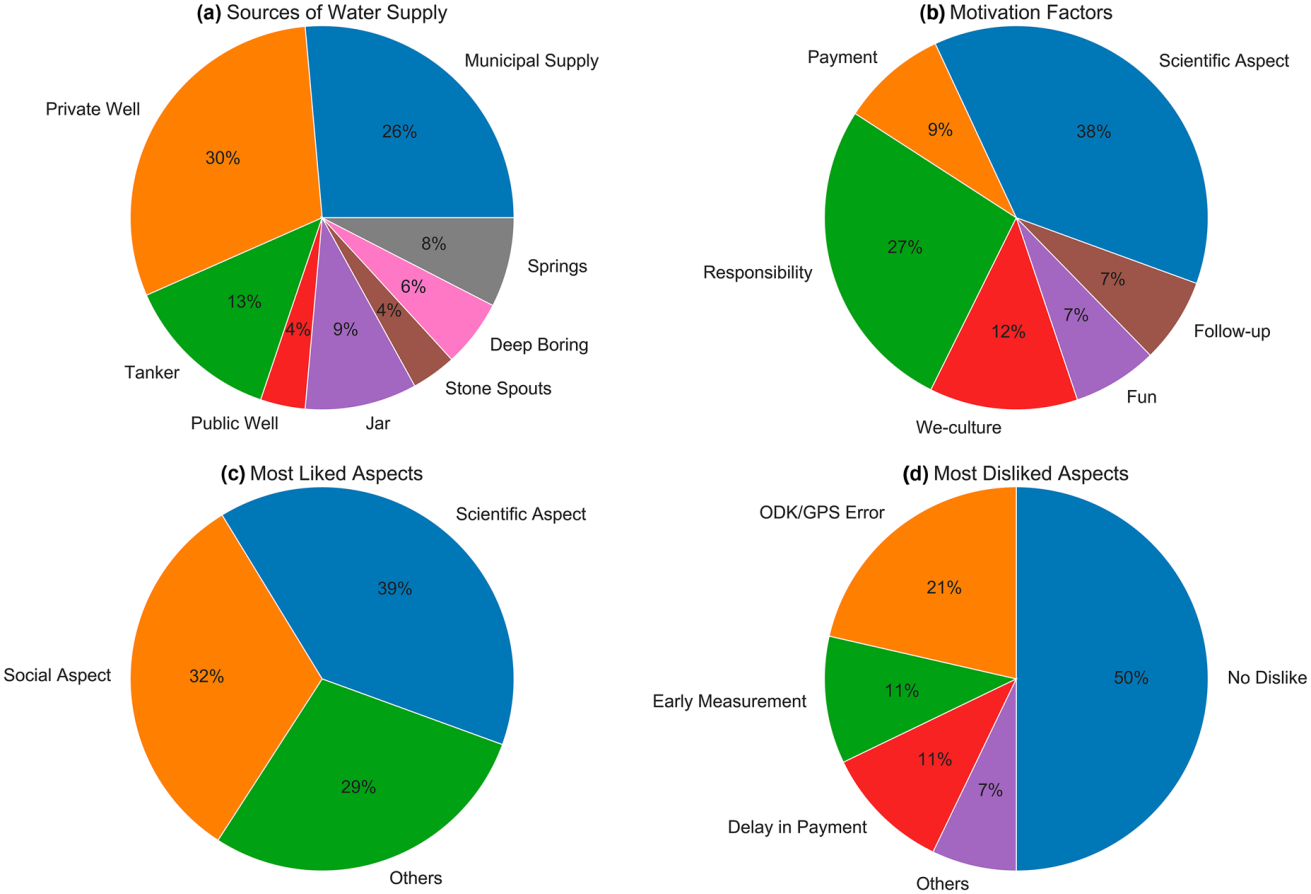
Results of December 2018–January 2019 citizen scientist survey showing **a** different sources of water supply near the monitoring sites (total number of responses (N) = 53), **b** various citizen scientists’ motivation factors (N = 56), **c** the most liked aspect of the study (N = 28), and **d** the most disliked aspect of the study (N = 28). For **a** and **b**, the respondents were allowed to choose multiple answers

Out of 45 groundwater monitoring sites, detailed information regarding the history of the existing wells, methods of groundwater extraction, the use of groundwater, land uses, and water supply status in the vicinity of the wells were collected from 34 of the sites. Nine (20%) of the monitoring wells were public wells and 36 (80%) were private. A majority of the public wells were more than 40 years old, while the private wells had an average age of 10 years. The oldest private well was 20 years old and the youngest 1 year old. Based on the time taken by citizen scientists to reach their respective monitoring wells, they were classified as nearby (less than a 5-min walking distance) and distant wells. Except for five monitoring sites, all wells were near the residence of citizen scientists responsible for data collection (e.g., a 5-min walk or less). Figure 4a indicates the major source of water supply in the vicinity of the monitoring sites. More than 75% of the locations were dependent on municipal supply and private wells. Citizen scientists noted that multiple sources are needed since the municipal supply was irregular and limited to 2–4 h per week. The use of jar water for drinking and tanker water for household purposes was common in urban centers. Groundwater from the monitoring wells was mostly used for household purposes such as food preparation and cleaning, washroom use, watering gardens or livestock, etc. Groundwater from only eight wells was used for drinking. Water extraction from the wells was accomplished by pumps for 14 (40%) monitoring wells, by hand using buckets for 10 (30%) wells, and by a combination of pumps and buckets for the remaining 10 (30%) wells.

### Recruitment of citizen scientists

A total of 45 citizen scientists were recruited to participate in the study. About 75% were recruited through personal connections, 22% through outreach programs at colleges, and only one (3%) through random field visits. Although frequent social media posts for volunteers helped publicize information to a larger audience, most citizen scientists were recruited through either personal connections or outreach programs at colleges. One-fifth of citizen scientists were female, and the rest were male. Except for two, all citizen scientists were between 20 and 25 years of age and had either recently completed or were currently enrolled in studies to obtain a Bachelor’s degree or were enrolled in their Bachelor’s studies. Roughly half of the citizen scientists were full-time students, and the rest were employed by either the government or the private sector.

### Citizen scientists’ motivation

Out of 45 citizen scientists, 28 (62%) participated in the online survey conducted in December 2018 – January 2019. The survey form included a question on the source of motivation for regular groundwater monitoring with the option of selecting multiple choices. A total of 21 respondents (75%) stated “Scientific aspect (Development of research skills, access to collected data)” as their major motivation, and 15 respondents (54%) chose “Responsibility as a Citizen Scientist” as one of their major motivations (Fig. 4b).

Survey results showed that the most liked aspect for citizen scientists was the scientific aspect of the monitoring program (Fig. 4c). The scientific aspect included education and experience in monitoring temporal groundwater-level fluctuations and their significance in the research. The social aspect of the monitoring program was the most liked aspect for roughly one-third of survey participants. The social aspect presented groundwater-level monitoring as social work to obtain valuable information for the local community. For survey participants who selected “Other” as their most liked aspect of the study, the two most popular most liked aspects were payment and regular follow-up from S4W-Nepal.

Regarding the most disliked aspect of the monitoring program, half of the participants stated there was nothing they disliked (Fig. 4d). The remaining participants indicated that errors with their smartphone app or GPS, early morning measurement times, delays in payments, the distance to monitoring well, or the frequent reminders for measurement and regular follow-up from S4W-Nepal were their most disliked aspects of the monitoring program.

More than 80% of respondents were interested in continuing groundwater monitoring for the next 3 years. Suggestions from citizen scientists for the improvement of the monitoring program included performing regular water quality monitoring in addition to groundwater-level monitoring, improved methods for dissemination of information generated from the collected data and its use, regular updates on the progress of the current program, and a need for frequent site visits from S4W-Nepal staff.

### Citizen scientist performance

**Fig. 5:**
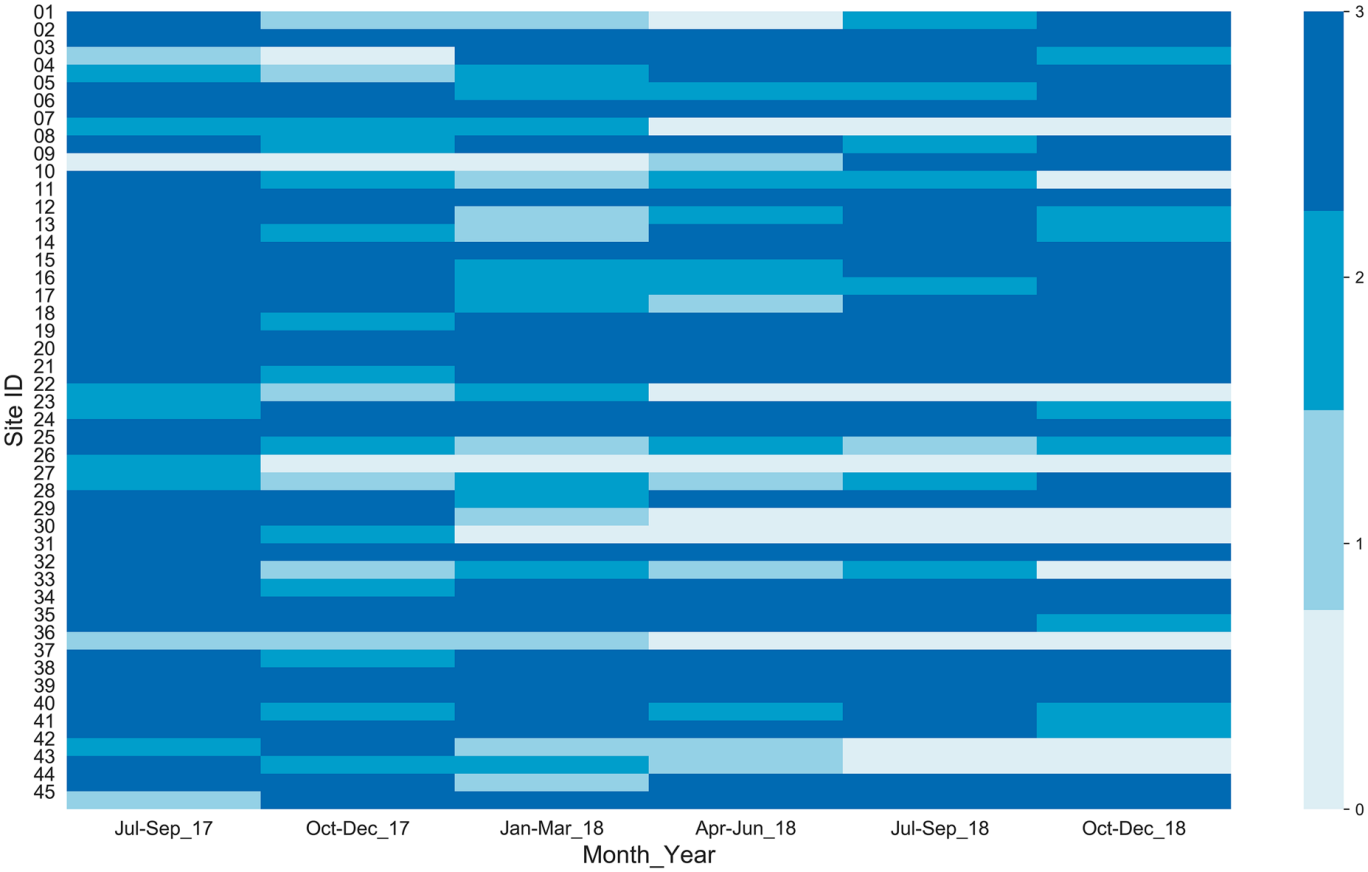
Heatmap showing the number of measurements per three months for the 18-month period from July 2017 through December 2018. Each column pixel represents three consecutive months and each row pixel represents an individual citizen scientist. The color of each pixel represents the number of measurements performed every 3 months. Light and dark blue represent one and three measurements, respectively; white means zero measurements were performed

Figure 5 depicts data collection trends for all 45 citizen scientists; citizen scientists who consistently participated in data collection are represented by continual dark blue rows while inconsistent citizen scientists are represented by rows with variations in blue color. Almost all citizen scientists were consistent in monthly measurements in the first 3 months (July–Sep 2017) but the frequency of measurements gradually decreased in subsequent months. The lowest number of measurements occurred in Jan–March 2018 and April–June 2018. The overall average of monthly measurements was 34, whereas the average monthly measurements for monsoon and non-monsoon seasons were 37 and 32, respectively, indicating more measurements during the monsoon period. In the monsoon season, the groundwater levels were shallow and fluctuations were more frequent than those in non-monsoon seasons. So, the citizen scientists seemed to take more frequent measurements in monsoon seasons when the groundwater fluctuations were more dynamic than non-monsoon seasons. This shows that citizen scientists tend to be more consistent in data collection in monsoon seasons, as compared to non-monsoon seasons. Altogether, 12 citizen scientists were consistent throughout the study period. Citizen scientists left the monitoring program for various reasons including loss of interest, moving out of the area, and loss of an android phone. Attempts to recruit additional citizen scientists to replace those who left were unsuccessful. Due to this, S4W-Nepal staff continued data collection at seven sites and ten monitoring sites were permanently discontinued (represented by consistent white color in Fig. 5). Also, improvements in follow-up and communication with citizen scientists from May 2018 onwards were effective in motivating the remaining citizen scientists to continue measurements and interestingly not as many citizen scientists left the program after that date. The highest number of measurements was recorded in August 2017 (43), whereas the least was recorded in March 2018 (25).

**Fig. 6:**
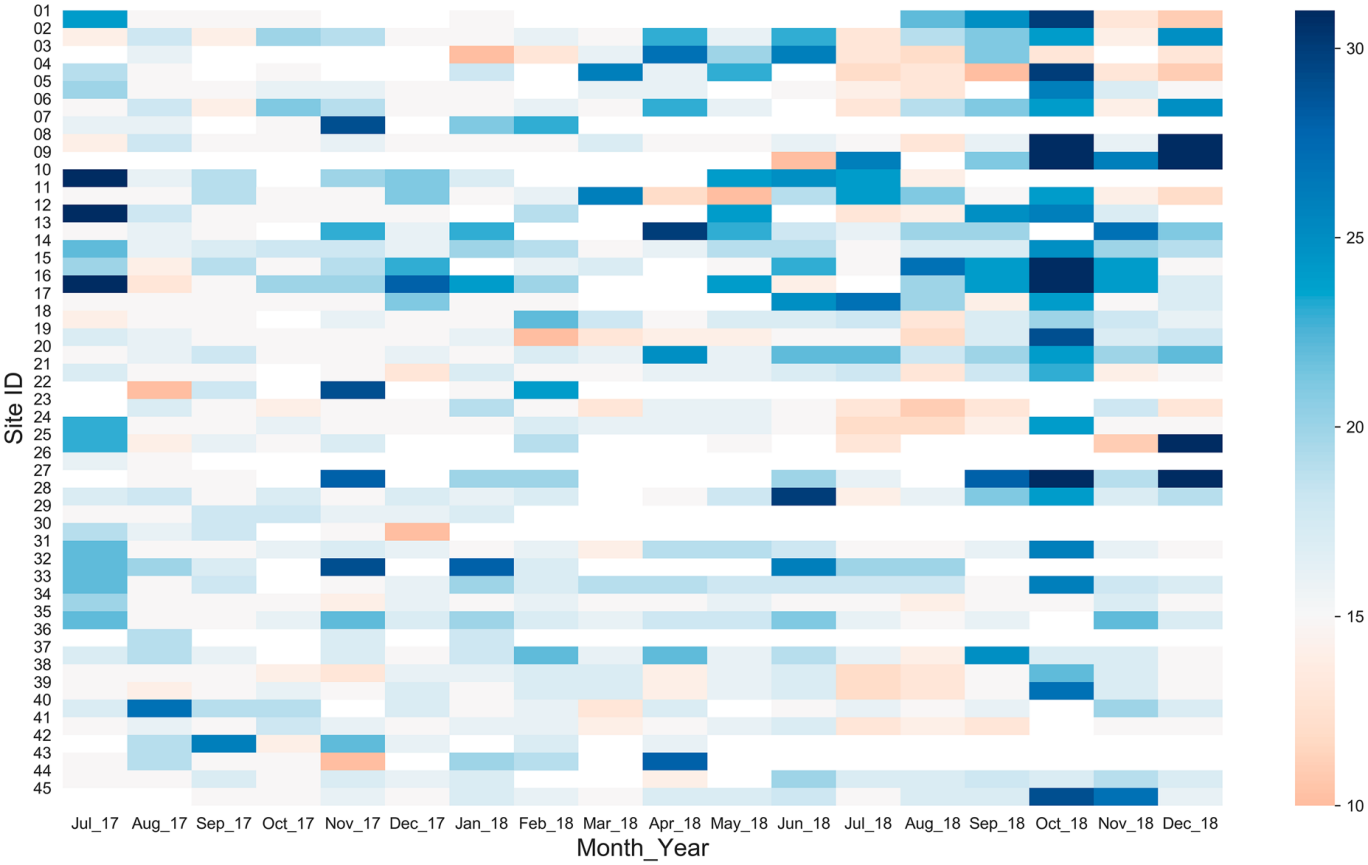
Heatmap showing the date of measurement in a particular month for an 18-month period from July 2017 through December 2018. Each column pixel represents a month and each row pixel represents an individual citizen scientist. The color of each pixel represents the date of measurement in the particular month. Orange and dark blue represent start and end of month, respectively; white means zero measurements were performed that month

Another heatmap (Fig. 6) illustrates the punctuality of citizen scientists’ measurements. As the measurements were performed by several citizen scientists, it is crucial that the data be taken on the same day each month so that the measurements were comparable to each other. As we aim to investigate on a monthly scale, we believe the 15th of the month (i.e., about a half of the month’s duration) was taken as a representation of the month. The target measurement date for each month was the 15th of the English calendar month which is generally the beginning of the Nepali calendar month, and it is quite easy for the citizen scientists to remember the date of measurement. The mean measurement date was the 17th with a standard deviation of roughly 4 days. The citizen scientists monitoring sites 21, 23, 34, 38, 40, and 44 were very punctual, consistently completing measurements by the 20th of the month, and the citizen scientists monitoring sites 4, 8, 19, 24, and 39 were almost as punctual. Figure 5 shows a delay in measurement at many sites in October 2018 and comparatively fewer measurements in October 2017, as compared to other months. This was due to the Dashain holiday, which occurs every October and is Nepal’s largest annual festival.

### Quantification of costs

S4W-Nepal allocated approximately 480 (2 staff * 30 days * 8 h per day) staff hours to recruit and train 45 citizen scientists and configuration of the monitoring network. All citizen scientists were provided with a standard 30-m measuring tape, $3.50USD each. After beginning data collection, S4W-Nepal allocated approximately 24 staff hours monthly to follow up with citizen scientists for regular measurements by voice call, SMS, and field visit if necessary (approximately $11USD monthly). Also, S4W-Nepal provided a small per observation transfer (approximately $1USD per citizen scientist) to their mobile phone account. Furthermore, we organized an annual workshop (approximately $300 per event) to disseminate the study findings to the citizen scientists. The overall costs are presented below in Table 1.

**Table 1:** Summary of initial configuration and monthly operational costs

**Cost category**	**Cost item**	**Unit**	**Quantity**	**Cost per unit (USD)**	**Total cost**
Initial configuration costs	Labor cost	Hours	480	$1	$480
	Measuring tape	Number	45	$3.50	$157.5
Total					$637.5
Monthly operational costs	Labor cost	Hours	24	$1	$24
	Communication charge	Number of citizen scientists	45	$0.25	$11
	Mobile transfer	Number of citizen scientists	45	$1	$45
	Measuring tape maintenance cost	LS			$5
	Workshop expense	LS			$25
**Total**					$110

The initial configuration cost per measurement and operational cost per measurement were calculated to be $14.50 and $2.50. In comparison, the equipment for an automated approach (i.e., pressure transducer) for a single monitoring site would cost approximately $900. Manual measurements by citizen scientists were preferred over an automated approach because of the cost savings and because pressure transducers are not readily available in the study area. Pressure transducers have the benefit of collecting continuous data at a regular interval (e.g., daily or hourly); however, since groundwater dynamics are usually slower, the information content of monthly measurements is similar to daily measurements. Citizen science-based monitoring also has additional benefits such as engaging and motivating local community members to care for local groundwater resources and disseminating the collected data to the local community and policymakers in an understandable format (Little et al., 2016).

### Groundwater level and dynamics

**Fig. 7:**
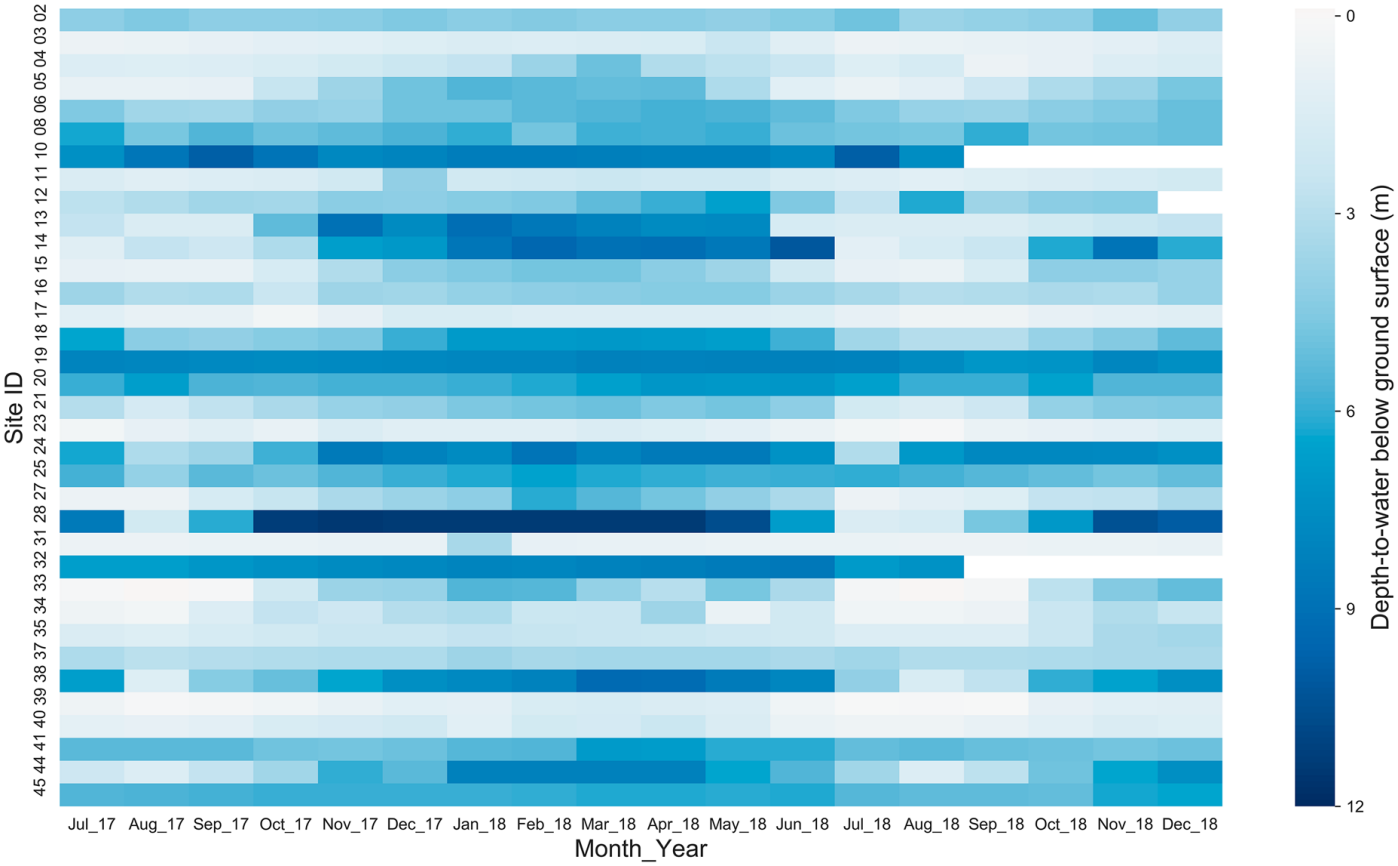
Heatmap showing the monthly groundwater depth-to-water from the ground surface for the 18-month period from July 2017 through December 2018. Each column pixel represents a month and each row pixel represents an individual citizen scientist. The color of each pixel represents the groundwater depth from ground surface in the particular month. Light and dark blue represent shallow and deep groundwater levels, respectively; white means zero measurements were performed that month. Sites 10 and 32 had zero measurements from September 2018 to December 2018

All groundwater-level data collected in various monitoring sites during the study period (July 2017 – December 2018) are publicly available. For the 35 wells with consistent data, over 18 months, the depth-to-water below the ground surface varied from − 0.11 m (negative sign represents a groundwater level higher than the ground surface) to 11.50 m, with a mean of 4.07 m and standard deviation of 2.63 m. Figure 7 illustrates groundwater-level fluctuations for all 35 monitoring sites. The monitoring wells whose surrounding land use were dominated by agricultural land use (sites 3, 23, 31, 39, and 40) had a shallow groundwater level (depth-to-water less than 2 m) throughout the study period. Most of the monitoring wells in built land use (sites 10, 19, and 32) had a depth-to-water of more than 6 m throughout the study period which might be due to a higher rate of groundwater extraction and sealed ground cover reducing groundwater recharge (Prajapati et al., 2021). Furthermore, the groundwater monitoring sites with land use covered by both agricultural and built type (sites 13, 14, 28, 38, and 44) had higher variability (greater than 6 m change), indicating the high seasonal fluctuation of groundwater levels in some areas. The highest change in groundwater levels was 9.95 m (site 28), and the lowest change was 0.85 m (site 37). Site 28 was dominated by built land use with excessive groundwater extraction, whereas site 37 was in agricultural land use with minimum extraction. Overall, the average groundwater level change for all sites was 3.51 m with a standard deviation of 2.39 m.

**Fig. 8:**
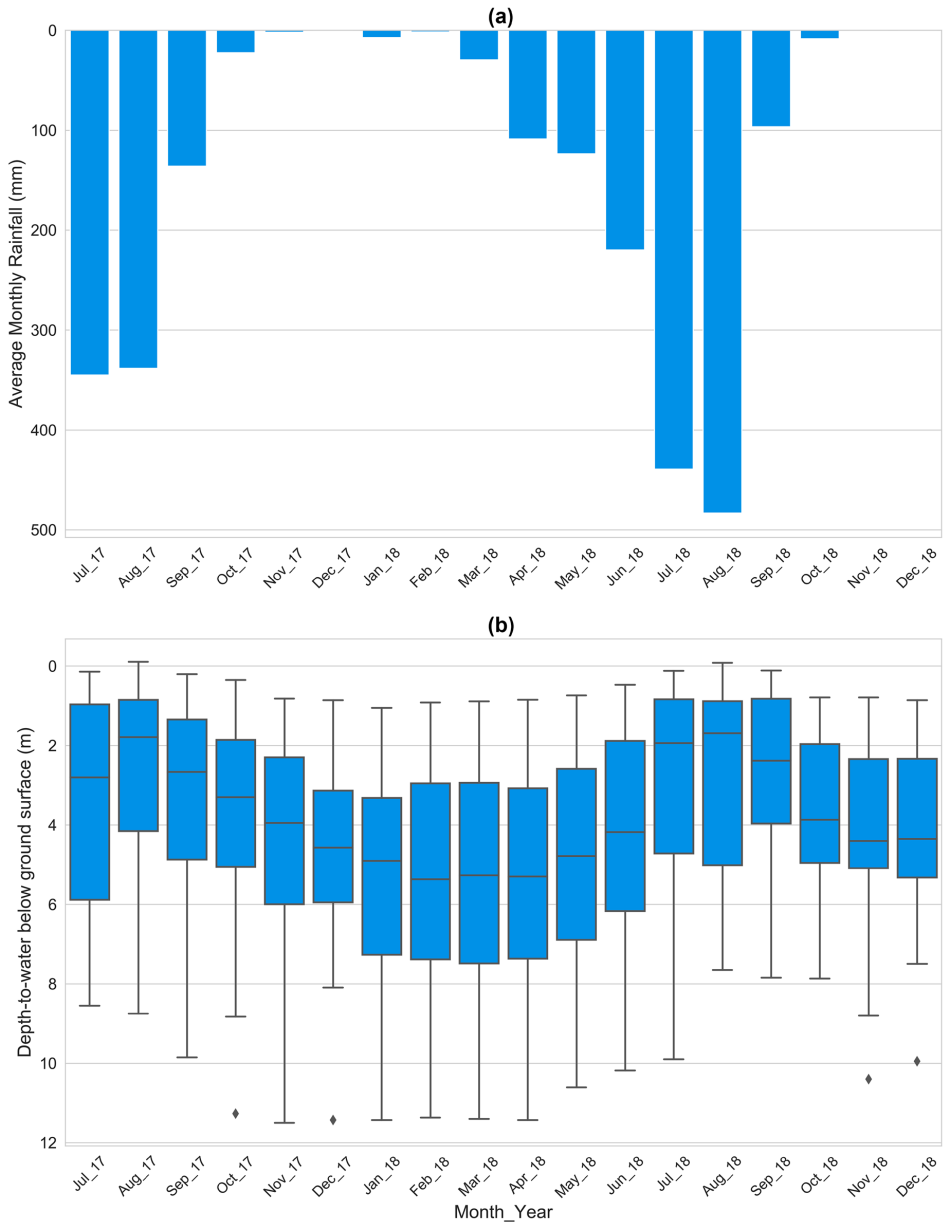
**a** Bar graph showing average monthly rainfall. **b** Box plot showing monthly median and distribution of groundwater depth measured from ground surface per month for all sites (n = 35 from July 2017 to August 2018 and n = 33 from September 2018 to December 2018)

**Fig. 9:**
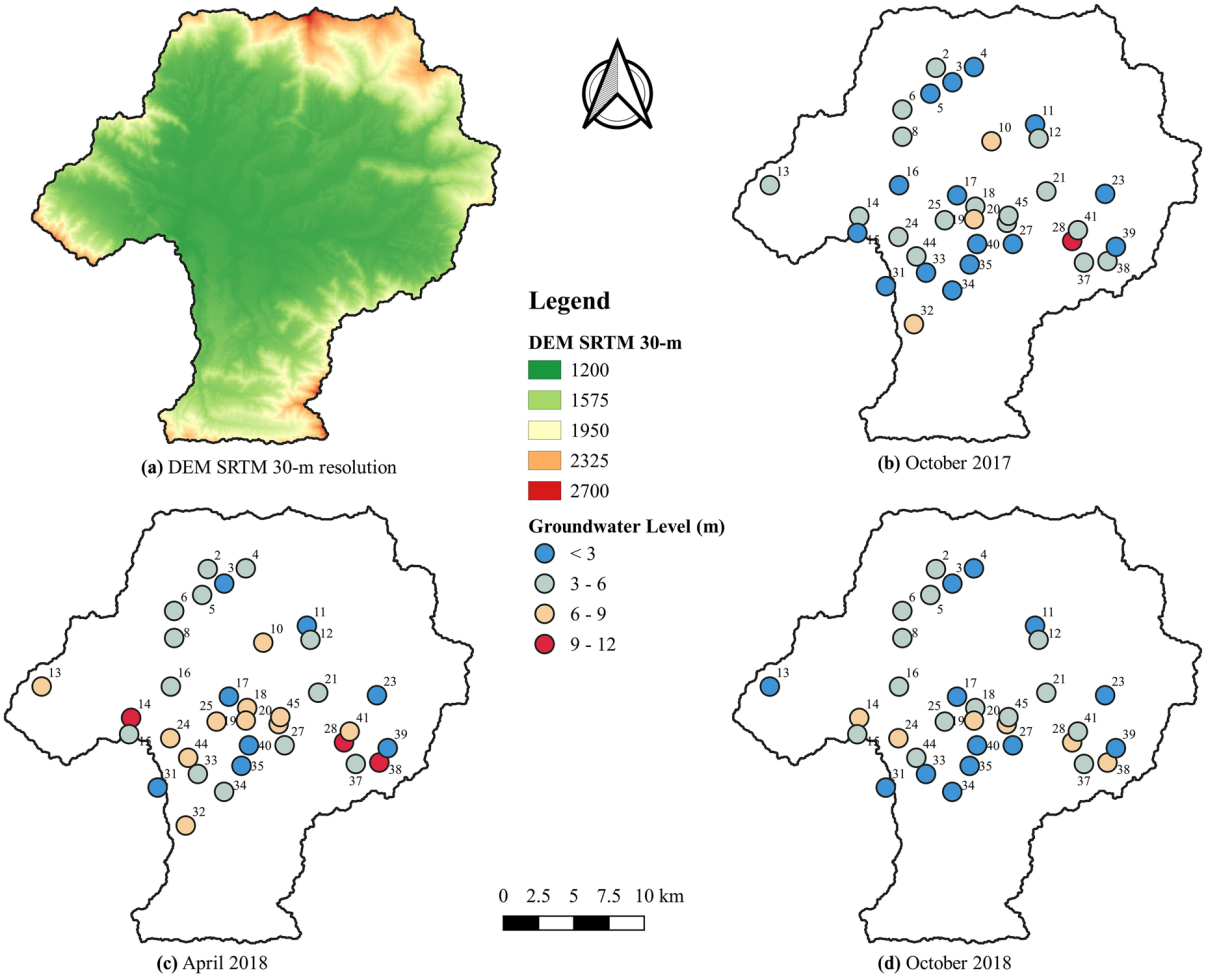
Kathmandu Valley watershed with **a** SRTM DEM at 30-m resolution, groundwater level depth (meter) below ground surface in **b** October 2017, **c** April 2017, and **d** October 2018

Figure 8a and b show the bar graph of average monthly rainfall and the box plot of all 35 wells over time, respectively. The mean annual rainfall was 1440 mm for the study period. The southern part received comparatively less rainfall than the northern part of the Valley (Prajapati et al., 2021). The groundwater recharge rate is high in monsoon as more than 80% of the annual rainfall occurs in the monsoon season. Groundwater levels began to rise abruptly with the onset of monsoon season (July) with the shallowest groundwater levels recorded in peak rainfall months (July and August). The deepest groundwater levels were recorded in dry months (February, March, and April) with the deepest overall month being February 2018. After the recharge signal from monsoon rainfall in July increased the median groundwater level, the median level then decreased from August through February because of dry conditions and continued groundwater extractions. Figure 9 shows Shuttle Radar Topography Mission (SRTM) Digital Elevation Model (DEM) at 30-m resolution, spatial maps of October 2017, April 2017, and October 2018. Spatial groundwater level fluctuation is mostly governed by nearby land uses at each monitoring location. The central part of the Valley, including the Kathmandu and Lalitpur metropolitan areas and the eastern part, including the Bhaktapur municipality, are mostly dominated by the built-high land use reflective of more intense urban development. The groundwater depth below the ground surface in these areas was greater than other parts of the Valley due to increased groundwater extraction and minimal recharge from surface sealing. The contribution of monsoon rainfall was substantial. In most parts of the Valley, groundwater levels in post-monsoon months (October 2017 and October 2018) were shallower (less than 3 m from the ground surface) as compared to the pre-monsoon month (April 2017) when levels were deeper (greater than 4 m from the ground surface). The groundwater levels in October 2017 and October 2018 were similar; differences seen were primarily caused by missing data in October 2018. The implications of variations in rainfall and land use groundwater-level fluctuations in the Valley were studied in detail by Prajapati et al. (2021).

### Outlook

One of the major advantages of the current research is the successful implementation of citizen science-based groundwater monitoring networks in the Valley. The study was able to generate baseline data which is inevitable for the shallow groundwater research in the Valley. It has already been used by researchers from different national and international universities and several government agencies for different purposes. Availability of smartphones and the internet to the general public, growing concerns in the youth regarding the conservation of water resources, availability of existing dug wells in the study area, etc., were the contributing factors of the project. The use of smartphone applications facilitated convenient data collection, transmission, quality control, and database management. In addition, the study used locally available low-cost measuring tape as monitoring equipment which significantly reduced the monitoring cost. The groundwater-level monitoring procedure, i.e., the use of smartphone application and measuring tape, was quite simple and took at most 15 minutes to learn. The simplicity and low-cost nature of the measurement technique proved crucial for the scalability in different circumstances.

The involvement of the general public in scientific research can increase the scope of data generation (both spatial and temporal) and enhance scientific awareness (Walker et al., 2021). The study engaged 45 citizen scientists of different backgrounds from several places of the Valley and helped improve their scientific knowledge regarding groundwater resources of the Valley. As most of the citizen scientists were students or recent graduates, “Scientific aspect (development of research skills, open access to collected data)” and “Responsibility as a Citizen Scientist” were their major motivations. Dissemination of collected data as valuable information in an understandable format to the citizen scientists and general public through different approaches (social media posts, web stories, etc.) on a regular basis was one of the important motivating factors.

Although leveraging personal connections was a cost-effective method for recruiting citizen scientists, it poses challenges to scalability (Davids et al., 2019). More scalable approaches (public outreaches in academic institutes, local communities, etc.) should be developed in future projects. The study was conducted in urban areas, and the targeted citizen scientists were mostly undergraduate students. So, the data generation approach may not be effective in rural areas with a limited student population and relatively low scientific literacy levels (Davids et al., 2019). Monetary incentives may be an alternative motivating factor in these rural areas (Davids et al., 2019). There was an uneven distribution of monitoring wells spatially due to the unavailability of existing dug wells in several locations. Setting up dedicated wells for monitoring was beyond the scope and budget of the study. Future efforts should explore the possible collaboration among different government and non-government agencies to establish new monitoring wells in those missing sites and work together for sustainable groundwater resource monitoring. Apart from monthly groundwater-level monitoring, seasonal groundwater quality monitoring is also recommended for the near future.

## Conclusions

We implemented a study in the Kathmandu Valley watershed to establish a citizen science-based groundwater monitoring network using existing wells, whereby local citizen scientists measure shallow groundwater levels in public or private wells of their community. The main features of the monitoring program were the close collaboration between young researchers and community volunteers (citizen scientists), and the integration of the monitoring program with education and outreach programs. Out of 45 total citizen scientists, twelve consistently participated throughout the study period and citizen scientists were more consistent in data collection during the monsoon season than other times of the year. We recruited 75% of citizen scientists through personal connections and used various methods to encourage citizen scientists for regular measurement such as reminder SMS/follow-up calls before measurement, distributing thank you messages, and a summary of monthly groundwater-level change through SMS after measurements, providing certificate of volunteering, providing access to data, organizing workshops, and offering payment.

The monitoring program has successfully generated data to better understand the seasonal groundwater-level dynamics in relation to natural and anthropogenic stresses, including rainfall and land use practice. Groundwater levels began to rise abruptly with the onset of monsoon season (July) and the shallowest groundwater levels were recorded in peak rainfall months (July and August), while the deepest groundwater levels were recorded in dry winter months (February, March, and April). The methodology of this study should be further evaluated in other similar contexts to see if they can be sustainably replicated to help fill other groundwater data and knowledge gaps. In addition to continuous long-term groundwater-level monitoring at a high spatial density, source and flow path identification, regular groundwater quality monitoring, and studies on the influence of rainfall, land use/cover, geology, groundwater extraction, and more are required for understanding the complex groundwater system.
